# Supplementary material for: Performance and Application of Commercially Available Loop-Mediated Isothermal Amplification (LAMP) Kits in Malaria Endemic and Non-Endemic Settings
Source: Diagnostics (Basel). 2021 Feb 18;11(2):336. doi: 10.3390/diagnostics11020336 (PMC7922894; doi:10.3390/diagnostics11020336)
Supplement: Supplementary file 1 [file diagnostics-11-00336-s001.pdf]

Methodology

A literature search was conducted in Pubmed.gov on 23rd Sept 2020 using the search terms (((LAMP) OR (Loop mediated isothermal amplification)) AND ((Malaria) OR (Plasmodium))).

There were in total 214 results. All 214 results were transferred to EndNote where the preliminary grouping of the publications took place. Out of the 214 results, in total 70 were excluded (27 were not about Loop-mediated isothermal amplification, 25 were not related to the detection of malaria in humans, 12 were not in English, 3 had no full text available; 3 case reports were also excluded). The remaining 144 publications were grouped based on commonly occurring key words and/or relevance (see below).

The list was continuously updated with new search results identified through weekly automatic Pubmed.gov search reports (using the same search terms as above) until the submission of the manuscript, together with any other articles of relevance identified during the writing process.

List of malaria LAMP publications, grouped by theme

Numbers in hard brackets = number of articles under each subheading.

Highlighted text = Which LAMP method was used in the publication, if not specified in the subheading

Malaria LAMP Reviews [12]

1. Abdul-Ghani R, Al-Mekhlafi AM, Karanis P. Loop-mediated isothermal amplification (LAMP) for malarial parasites of humans: would it come to clinical reality as a point-of-care test? Acta Trop 2012, 122:233-40.
2. Han ET. Loop-mediated isothermal amplification test for the molecular diagnosis of malaria. Expert Rev Mol Diagn 2013, 13:205-18.
3. Oriero EC, Jacobs J, Van Geertruyden JP, Nwakanma D, D'Alessandro U. Molecular-based isothermal tests for field diagnosis of malaria and their potential contribution to malaria elimination. J Antimicrob Chemother 2015, 70:2-13.
4. Oriero EC, Van Geertruyden JP, Nwakanma DC, D'Alessandro U, Jacobs J. Novel techniques and future directions in molecular diagnosis of malaria in resource-limited settings. Expert Rev Mol Diagn 2015, 15:1419-26.
5. Britton S, Cheng Q, McCarthy JS. Novel molecular diagnostic tools for malaria elimination: a review of options from the point of view of high-throughput and applicability in resource limited settings. Malar J 2016, 15:88.
6. Amir A, Cheong FW, De Silva JR, Lau YL. Diagnostic tools in childhood malaria. Parasit Vectors 2018, 11:53.
7. Gruenberg M, Moniz CA, Hofmann NE, Wampfler R, Koepfli C, Mueller I, et al. Plasmodium vivax molecular diagnostics in community surveys: pitfalls and solutions. Malar J 2018, 17:55.
8. Lucchi NW, Ndiaye D, Britton S, Udhayakumar V. Expanding the malaria molecular diagnostic options: opportunities and challenges for loop-mediated isothermal amplification tests for malaria control and elimination. Expert Rev Mol Diagn 2018, 18:195-203.
9. Mukkala AN, Kwan J, Lau R, Harris D, Kain D, Boggild AK. An Update on Malaria Rapid Diagnostic Tests. Curr Infect Dis Rep 2018, 20:49.
10. Mbanefo A, Kumar N. Evaluation of Malaria Diagnostic Methods as a Key for Successful Control and Elimination Programs. Trop Med Infect Dis 2020, 5.

11. Varo R, Balanza N, Mayor A, Bassat Q. Diagnosis of clinical malaria in endemic settings. Expert Rev Anti Infect Ther 2020:1-14.
12. Zainabadi K: Ultrasensitive diagnostics for low density asymptomatic Plasmodium falciparum infections in low transmission settings. *J Clin Microbiol* 2020.

Systematic reviews and meta-analysis [3]

13. Roth JM, Korevaar DA, Leeflang MM, Mens PF. Molecular malaria diagnostics: A systematic review and meta-analysis. Crit Rev Clin Lab Sci 2016, 53:87-105.
14. Picot S, Cucherat M, Bienvenu AL. Systematic review and meta-analysis of diagnostic accuracy of loop-mediated isothermal amplification (LAMP) methods compared with microscopy, polymerase chain reaction and rapid diagnostic tests for malaria diagnosis. Int J Infect Dis 2020, 98:408-19.
15. Selvarajah D, Naing C, Htet NH, Mak JW. Loop-mediated isothermal amplification (LAMP) test for diagnosis of uncomplicated malaria in endemic areas: a meta-analysis of diagnostic test accuracy. Malar J 2020, 19:211.

Malaria LAMP Editorials and correspondences [3]

16. Hsiang MS, Greenhouse B, Rosenthal PJ. Point of care testing for malaria using LAMP, loop mediated isothermal amplification. J Infect Dis 2014, 210:1167-9.
17. Chiodini PL. LAMP in the context of travellers' malaria: a shining light? Travel Med Infect Dis 2015, 13:126-7.
18. Goyal K, Kaur H, Sehgal A, Sehgal R. RealAmp Loop-Mediated Isothermal Amplification as a Point-of-Care Test for Diagnosis of Malaria: Neither Too Close nor Too Far. J Infect Dis 2015, 211:1686.

Non-commercialised LAMP methods

Early malaria LAMP development [5]

19. Poon LL, Wong BW, Ma EH, Chan KH, Chow LM, Abeyewickreme W, et al. Sensitive and inexpensive molecular test for falciparum malaria: detecting Plasmodium falciparum DNA directly from heat-treated blood by loop-mediated isothermal amplification. Clin Chem 2006, 52:303-6.
20. Han ET, Watanabe R, Sattabongkot J, Khuntirat B, Sirichaisinthop J, Iriko H, et al. Detection of four Plasmodium species by genus- and species-specific loop-mediated isothermal amplification for clinical diagnosis. J Clin Microbiol 2007, 45:2521-8.
21. Paris DH, Imwong M, Faiz AM, Hasan M, Yunus EB, Silamut K, et al. Loop-mediated isothermal PCR (LAMP) for the diagnosis of falciparum malaria. Am J Trop Med Hyg 2007, 77:972-6.
22. Yamamura M, Makimura K, Ota Y. Evaluation of a new rapid molecular diagnostic system for Plasmodium falciparum combined with DNA filter paper, loop-mediated isothermal amplification, and melting curve analysis. Jpn J Infect Dis 2009, 62:20-5.
23. Polley SD, Mori Y, Watson J, Perkins MD, González IJ, Notomi T, et al. Mitochondrial DNA targets increase sensitivity of malaria detection using loop-mediated isothermal amplification. J Clin Microbiol 2010, 48:2866-71.

Real-time detection platforms [2]

24. Lucchi NW, Demas A, Narayanan J, Sumari D, Kabanywanyi A, Kachur SP, et al. Real-time fluorescence loop mediated isothermal amplification for the diagnosis of malaria. PLoS One 2010, 5:e13733.
25. Surabattula R, Vejandla MP, Mallepaddi PC, Faulstich K, Polavarapu R. Simple, rapid, inexpensive platform for the diagnosis of malaria by loop mediated isothermal amplification (LAMP). Exp Parasitol 2013, 134:333-40.

Alternative extraction and extraction-free methods [4]

26. Port JR, Nguetse C, Adukpo S, Velavan TP. A reliable and rapid method for molecular detection of malarial parasites using microwave irradiation and loop mediated isothermal amplification. Malar J 2014, 13:454.
27. Modak SS, Barber CA, Geva E, Abrams WR, Malamud D, Ongagna YS. Rapid Point-of-Care Isothermal Amplification Assay for the Detection of Malaria without Nucleic Acid Purification. Infect Dis (Auckl) 2016, 9:1-9.
28. Xu G, Nolder D, Reboud J, Oguike MC, van Schalkwyk DA, Sutherland CJ, et al. Paper-Origami-Based Multiplexed Malaria Diagnostics from Whole Blood. Angew Chem Int Ed Engl 2016, 55:15250-3.
29. Hayashida K, Kajino K, Simukoko H, Simuunza M, Ndebe J, Chota A, et al. Direct detection of falciparum and non-falciparum malaria DNA from a drop of blood with high sensitivity by the dried-LAMP system. Parasit Vectors 2017, 10:26.

Alternative dyes for improved visualization of results [5]

30. Mohon AN, Elahi R, Khan WA, Haque R, Sullivan DJ, Jr., Alam MS. A new visually improved and sensitive loop mediated isothermal amplification (LAMP) for diagnosis of symptomatic falciparum malaria. Acta Trop 2014, 134:52-7.
31. Lucchi NW, Ljolje D, Silva-Flannery L, Udhayakumar V. Use of Malachite Green-Loop Mediated Isothermal Amplification for Detection of Plasmodium spp. Parasites. PLoS One 2016, 11:e0151437.
32. Singh R, Singh DP, Savargaonkar D, Singh OP, Bhatt RM, Valecha N. Evaluation of SYBR green I based visual loop-mediated isothermal amplification (LAMP) assay for genus and species-specific diagnosis of malaria in P. vivax and P. falciparum endemic regions. J Vector Borne Dis 2017, 54:54-60.
33. Barazorda KA, Salas CJ, Bishop DK, Lucchi N, Valdivia HO. Comparison of real time and malachite-green based loop-mediated isothermal amplification assays for the detection of Plasmodium vivax and P. falciparum. PLoS One 2020, 15:e0234263.
34. Lai MY, Ooi CH, Jaimin JJ, Lau YL. Evaluation of WarmStart Colorimetric Loop-Mediated Isothermal Amplification Assay for Diagnosis of Malaria. Am J Trop Med Hyg 2020, 102:1370-2.

Lateral flow dipstick [3]

35. Yongkiettrakul S, Jaroenram W, Arunrut N, Chareanchim W, Pannengpetch S, Suebsing R, et al. Application of loop-mediated isothermal amplification assay combined with lateral flow dipstick for detection of Plasmodium falciparum and Plasmodium vivax. Parasitol Int 2014, 63:777-84.
36. Kongkasuriyachai D, Yongkiettrakul S, Kiatpathomchai W, Arunrut N. Loop-Mediated Isothermal Amplification and LFD Combination for Detection of Plasmodium falciparum and Plasmodium vivax. Methods Mol Biol 2017, 1572:431-43.

37. Mallepaddi PC, Lai MY, Podha S, Ooi CH, Liew JW, Polavarapu R, et al. Development of Loop-Mediated Isothermal Amplification-Based Lateral Flow Device Method for the Detection of Malaria. Am J Trop Med Hyg 2018, 99:704-8.

High-throughput platforms [1; see also Loopamp MALARIA kit (Eiken)]

38. Britton S, Cheng Q, Sutherland CJ, McCarthy JS. A simple, high-throughput, colourimetric, field applicable loop-mediated isothermal amplification (HtLAMP) assay for malaria elimination. Malar J 2015, 14:335.

Ultrasensitive LAMP method [1]

39. Mohon AN, Getie S, Jahan N, Alam MS, Pillai DR. Ultrasensitive loop mediated isothermal amplification (US-LAMP) to detect malaria for elimination. Malar J 2019, 18:350.

Alternative biological materials [2]

40. Ghayour Najafabadi Z, Oormazdi H, Akhlaghi L, Meamar AR, Nateghpour M, Farivar L, et al. Detection of Plasmodium vivax and Plasmodium falciparum DNA in human saliva and urine: loop-mediated isothermal amplification for malaria diagnosis. Acta Trop 2014, 136:44-9.
41. Hashimoto M, Sakamoto H, Ido Y, Tanaka M, Yatsushiro S, Kajimoto K, et al. In situ loop-mediated isothermal amplification (LAMP) for identification of Plasmodium species in wide-range thin blood smears. Malar J 2018, 17:235.

Other non-commercial LAMP methods [10]

42. Oriero CE, van Geertruyden JP, Jacobs J, D'Alessandro U, Nwakanma D. Validation of an apicoplast genome target for the detection of Plasmodium species using polymerase chain reaction and loop mediated isothermal amplification. Clin Microbiol Infect 2015, 21:686.e1-7.
43. Choi G, Song D, Shrestha S, Miao J, Cui L, Guan W. A field-deployable mobile molecular diagnostic system for malaria at the point of need. Lab Chip 2016, 16:4341-9.
44. Kemleu S, Guelig D, Eboumbou Moukoko C, Essangui E, Diesburg S, Mouliom A, et al. A Field-Tailored Reverse Transcription Loop-Mediated Isothermal Assay for High Sensitivity Detection of Plasmodium falciparum Infections. PLoS One 2016, 11:e0165506.
45. Imai K, Tarumoto N, Misawa K, Runtuwene LR, Sakai J, Hayashida K, et al. A novel diagnostic method for malaria using loop-mediated isothermal amplification (LAMP) and MinION™ nanopore sequencer. BMC Infect Dis 2017, 17:621.
46. Zhang Y, Yao Y, Du W, Wu K, Xu W, Lin M, et al. Development of loop-mediated isothermal amplification with Plasmodium falciparum unique genes for molecular diagnosis of human malaria. Pathog Glob Health 2017, 111:247-55.
47. Choi G, Prince T, Miao J, Cui L, Guan W. Sample-to-answer palm-sized nucleic acid testing device towards low-cost malaria mass screening. Biosens Bioelectron 2018, 115:83-90.
48. Kadimisetty K, Song J, Doto AM, Hwang Y, Peng J, Mauk MG, et al. Fully 3D printed integrated reactor array for point-of-care molecular diagnostics. Biosens Bioelectron 2018, 109:156-63.
49. Mao R, Ge G, Wang Z, Hao R, Zhang G, Yang Z, et al. A multiplex microfluidic loop-mediated isothermal amplification array for detection of malaria-related parasites and vectors. Acta Trop 2018, 178:86-92.
50. Aninagyei E, Smith-Graham S, Boye A, Egyir-Yawson A, Acheampong DO. Evaluating 18s-rRNA LAMP and selective whole genome amplification (sWGA) assay in detecting asymptomatic Plasmodium falciparum infections in blood donors. Malar J 2019, 18:214.

51. Tang Z, Choi G, Nouri R, Guan W. Loop-Mediated Isothermal Amplification-Coupled Glass Nanopore Counting Toward Sensitive and Specific Nucleic Acid Testing. Nano Lett 2019, 19:7927-34.

Species specific LAMP methods

P. vivax LAMP [9]

52. Chen JH, Lu F, Lim CS, Kim JY, Ahn HJ, Suh IB, et al. Detection of Plasmodium vivax infection in the Republic of Korea by loop-mediated isothermal amplification (LAMP). Acta Trop 2010, 113:61-5.
53. Tao ZY, Zhou HY, Xia H, Xu S, Zhu HW, Culleton RL, et al. Adaptation of a visualized loop-mediated isothermal amplification technique for field detection of Plasmodium vivax infection. Parasit Vectors 2011, 4:115.
54. Lu F, Gao Q, Zhou H, Cao J, Wang W, Lim CS, et al. Molecular test for vivax malaria with loop-mediated isothermal amplification method in central China. Parasitol Res 2012, 110:2439-44.
55. Patel JC, Oberstaller J, Xayavong M, Narayanan J, DeBarry JD, Srinivasamoorthy G, et al. Real-time loop-mediated isothermal amplification (RealAmp) for the species-specific identification of Plasmodium vivax. PLoS One 2013, 8:e54986.
56. Singh R, Savargaonkar D, Bhatt R, Valecha N. Rapid detection of Plasmodium vivax in saliva and blood using loop mediated isothermal amplification (LAMP) assay. J Infect 2013, 67:245-7.
57. Dinzouna-Boutamba SD, Yang HW, Joo SY, Jeong S, Na BK, Inoue N, et al. The development of loop-mediated isothermal amplification targeting alpha-tubulin DNA for the rapid detection of Plasmodium vivax. Malar J 2014, 13:248.
58. Kim JY, Goo YK, Ji SY, Shin HI, Han ET, Hong Y, et al. Development and efficacy of real-time PCR in the diagnosis of vivax malaria using field samples in the Republic of Korea. PLoS One 2014, 9:e105871.
59. Britton S, Cheng Q, Grigg MJ, Poole CB, Pasay C, William T, et al. Sensitive Detection of Plasmodium vivax Using a High-Throughput, Colourimetric Loop Mediated Isothermal Amplification (HtLAMP) Platform: A Potential Novel Tool for Malaria Elimination. PLoS Negl Trop Dis 2016, 10:e0004443.
60. Kaur H, Sehgal R, Bansal D, Sultan AA, Bhalla A, Singhi SC. Development of Visually Improved Loop Mediated Isothermal Amplification for the Diagnosis of Plasmodium vivax Malaria in a Tertiary Hospital in Chandigarh, North India. Am J Trop Med Hyg 2018, 98:1374-81.

P. Knowelsi LAMP [3]

61. Iseki H, Kawai S, Takahashi N, Hirai M, Tanabe K, Yokoyama N, et al. Evaluation of a loop-mediated isothermal amplification method as a tool for diagnosis of infection by the zoonotic simian malaria parasite Plasmodium knowlesi. J Clin Microbiol 2010, 48:2509-14.
62. Lau YL, Fong MY, Mahmud R, Chang PY, Palaeya V, Cheong FW, et al. Specific, sensitive and rapid detection of human plasmodium knowlesi infection by loop-mediated isothermal amplification (LAMP) in blood samples. Malar J 2011, 10:197.
63. Britton S, Cheng Q, Grigg MJ, William T, Anstey NM, McCarthy JS. A Sensitive, Colorimetric, High-Throughput Loop-Mediated Isothermal Amplification Assay for the Detection of Plasmodium knowlesi. Am J Trop Med Hyg 2016, 95:120-2.

Other species-specific LAMP methods [2]

64. Kollenda H, Hagen RM, Hanke M, Rojak S, Hinz R, Wassill L, et al. Poor Diagnostic Performance of a Species-Specific Loop-Mediated Isothermal Amplification (LAMP) Platform for Malaria. Eur J Microbiol Immunol (Bp) 2018, 8:112-8.
65. Kristan M, Thorburn SG, Hafalla JC, Sutherland CJ, Oguike MC. Mosquito and human hepatocyte infections with Plasmodium ovale curtisi and Plasmodium ovale wallikeri. Trans R Soc Trop Med Hyg 2019, 113:617-22.

Evaluation of non-commercial LAMP methods in field settings [12]

66. Pöschl B, Waneesorn J, Thekisoe O, Chutipongvivate S, Karanis P. Comparative diagnosis of malaria infections by microscopy, nested PCR, and LAMP in northern Thailand. Am J Trop Med Hyg 2010, 83:56-60.
67. Sirichaisinthop J, Buates S, Watanabe R, Han ET, Suktawonjaroenpon W, Krasaesub S, et al. Evaluation of loop-mediated isothermal amplification (LAMP) for malaria diagnosis in a field setting. Am J Trop Med Hyg 2011, 85:594-6.
68. Lee PW, Ji DD, Liu CT, Rampao HS, do Rosario VE, Lin IF, et al. Application of loop-mediated isothermal amplification for malaria diagnosis during a follow-up study in São Tomé. Malar J 2012, 11:408.
69. Patel JC, Lucchi NW, Srivastava P, Lin JT, Sug-Aram R, Aruncharus S, et al. Field evaluation of a real-time fluorescence loop-mediated isothermal amplification assay, RealAmp, for the diagnosis of malaria in Thailand and India. J Infect Dis 2014, 210:1180-7.
70. Sattabongkot J, Tsuboi T, Han ET, Bantuchai S, Buates S. Loop-mediated isothermal amplification assay for rapid diagnosis of malaria infections in an area of endemicity in Thailand. J Clin Microbiol 2014, 52:1471-7.
71. Oriero EC, Okebe J, Jacobs J, Van Geertruyden JP, Nwakanma D, D'Alessandro U. Diagnostic performance of a novel loop-mediated isothermal amplification (LAMP) assay targeting the apicoplast genome for malaria diagnosis in a field setting in sub-Saharan Africa. Malar J 2015, 14:396.
72. Lau YL, Lai MY, Fong MY, Jelip J, Mahmud R. Loop-Mediated Isothermal Amplification Assay for Identification of Five Human Plasmodium Species in Malaysia. Am J Trop Med Hyg 2016, 94:336-9.
73. Ocker R, Prompunjai Y, Chutipongvivate S, Karanis P. MALARIA DIAGNOSIS BY LOOP-MEDIATED ISOTHERMAL AMPLIFICATION (LAMP) IN THAILAND. Rev Inst Med Trop Sao Paulo 2016, 58:27.
74. Viana GMR, Silva-Flannery L, Lima Barbosa DR, Lucchi N, do Valle SCN, Farias S, et al. Field evaluation of a real time loop-mediated isothermal amplification assay (RealAmp) for malaria diagnosis in Cruzeiro do Sul, Acre, Brazil. PLoS One 2018, 13:e0200492.
75. Girma S, Cheaveau J, Mohon AN, Marasinghe D, Legese R, Balasingam N, et al. Prevalence and Epidemiological Characteristics of Asymptomatic Malaria Based on Ultrasensitive Diagnostics: A Cross-sectional Study. Clin Infect Dis 2019, 69:1003-10.
76. Kudyba HM, Louzada J, Ljolje D, Kudyba KA, Muralidharan V, Oliveira-Ferreira J, et al. Field evaluation of malaria malachite green loop-mediated isothermal amplification in health posts in Roraima state, Brazil. Malar J 2019, 18:98.
77. Gachugia J, Chebore W, Otieno K, Ngugi CW, Godana A, Kariuki S. Evaluation of the colorimetric malachite green loop-mediated isothermal amplification (MG-LAMP) assay for the detection of malaria species at two different health facilities in a malaria endemic area of western Kenya. Malar J 2020, 19:329.

Commercial LAMP kits

Evaluation of Illumina platform [1; See also LAMP in malaria and LAMP in travellers]

78. Lucchi NW, Gaye M, Diallo MA, Goldman IF, Ljolje D, Deme AB, et al. Evaluation of the Illumigene Malaria LAMP: A Robust Molecular Diagnostic Tool for Malaria Parasites. Sci Rep 2016, 6:36808.

Loopamp MALARIA kit (Eiken Chemical Co)

Evaluation of Eiken Loopamp MALARIA kit in laboratory settings [4; see also "High through put extraction.." and "…detection of non-falciparum spp"]

79. Aydin-Schmidt B, Xu W, González IJ, Polley SD, Bell D, Shakely D, et al. Loop mediated isothermal amplification (LAMP) accurately detects malaria DNA from filter paper blood samples of low density parasitaemias. PLoS One 2014, 9:e103905.
80. Polley SD, Bell D, Oliver J, Tully F, Perkins MD, Chiodini PL, et al. The design and evaluation of a shaped filter collection device to sample and store defined volume dried blood spots from finger pricks. Malar J 2015, 14:45.
81. Vincent JP, Komaki-Yasuda K, Iwagami M, Kawai S, Kano S. Combination of PURE-DNA extraction and LAMP-DNA amplification methods for accurate malaria diagnosis on dried blood spots. Malar J 2018, 17:373.
82. Serra-Casas E, Guetens P, Chiheb D, Gamboa D, Rosanas-Urgell A. A pilot evaluation of alternative procedures to simplify LAMP-based malaria diagnosis in field conditions. Acta Trop 2019, 200:105125.

Evaluation of Eiken Loopamp MALARIA kit in field settings [10; see also "High through put extraction..", "…detection of non-falciparum spp" and "LAMP in Pregnancy"]

83. Hopkins H, González IJ, Polley SD, Angutoko P, Ategeka J, Asiimwe C, et al. Highly sensitive detection of malaria parasitemia in a malaria-endemic setting: performance of a new loop-mediated isothermal amplification kit in a remote clinic in Uganda. J Infect Dis 2013, 208:645-52.
84. Cook J, Aydin-Schmidt B, González IJ, Bell D, Edlund E, Nassor MH, et al. Loop-mediated isothermal amplification (LAMP) for point-of-care detection of asymptomatic low-density malaria parasite carriers in Zanzibar. Malar J 2015, 14:43.
85. Cuadros J, Pérez-Tanoira R, Prieto-Pérez L, Martin-Martin I, Berzosa P, González V, et al. Field Evaluation of Malaria Microscopy, Rapid Malaria Tests and Loop-Mediated Isothermal Amplification in a Rural Hospital in South Western Ethiopia. PLoS One 2015, 10:e0142842.
86. Morris U, Khamis M, Aydin-Schmidt B, Abass AK, Msellem MI, Nassor MH, et al. Field deployment of loop-mediated isothermal amplification for centralized mass-screening of asymptomatic malaria in Zanzibar: a pre-elimination setting. Malar J 2015, 14:205.
87. Sema M, Alemu A, Bayih AG, Getie S, Getnet G, Guelig D, et al. Evaluation of non-instrumented nucleic acid amplification by loop-mediated isothermal amplification (NINA-LAMP) for the diagnosis of malaria in Northwest Ethiopia. Malar J 2015, 14:44.
88. Katrak S, Murphy M, Nayebare P, Rek J, Smith M, Arinaitwe E, et al. Performance of Loop-Mediated Isothermal Amplification for the Identification of Submicroscopic Plasmodium falciparum Infection in Uganda. Am J Trop Med Hyg 2017, 97:1777-81.
89. Serra-Casas E, Manrique P, Ding XC, Carrasco-Escobar G, Alava F, Gave A, et al. Loop-mediated isothermal DNA amplification for asymptomatic malaria detection in challenging field settings:

Technical performance and pilot implementation in the Peruvian Amazon. PLoS One 2017, 12:e0185742.
90. Tambo M, Auala JR, Sturrock HJ, Kleinschmidt I, Bock R, Smith JL, et al. Evaluation of loop-mediated isothermal amplification as a surveillance tool for malaria in reactive case detection moving towards elimination. Malar J 2018, 17:255.
91. Mhamilawa LE, Aydin-Schmidt B, Mmbando BP, Ngasala B, Morris U. Detection of Plasmodium falciparum by Light Microscopy, Loop-Mediated Isothermal Amplification, and Polymerase Chain Reaction on Day 3 after Initiation of Artemether-Lumefantrine Treatment for Uncomplicated Malaria in Bagamoyo District, Tanzania: A Comparative Trial. Am J Trop Med Hyg 2019, 101:1144-7.
92. Hsiang MS, Ntshalintshali N, Kang Dufour MS, Dlamini N, Nhlabathi N, Vilakati S, et al. Active Case Finding for Malaria: A 3-Year National Evaluation of Optimal Approaches to Detect Infections and Hotspots Through Reactive Case Detection in the Low-transmission Setting of Eswatini. Clin Infect Dis 2020, 70:1316-25.

High through put extraction coupled with Eiken Loopamp MALARIA kit [2]

93. Aydin-Schmidt B, Morris U, Ding XC, Jovel I, Msellem MI, Bergman D, et al. Field Evaluation of a High Throughput Loop Mediated Isothermal Amplification Test for the Detection of Asymptomatic Plasmodium Infections in Zanzibar. PLoS One 2017, 12:e0169037.
94. Perera RS, Ding XC, Tully F, Oliver J, Bright N, Bell D, et al. Development and clinical performance of high throughput loop-mediated isothermal amplification for detection of malaria. PLoS One 2017, 12:e0171126.

Eiken Loopamp MALARIA kit for detection of non-falciparum spp. [5]

95. Vallejo AF, Martínez NL, González IJ, Arévalo-Herrera M, Herrera S. Evaluation of the loop mediated isothermal DNA amplification (LAMP) kit for malaria diagnosis in P. vivax endemic settings of Colombia. PLoS Negl Trop Dis 2015, 9:e3453.
96. Cuadros J, Martin Ramírez A, González IJ, Ding XC, Perez Tanoira R, Rojo-Marcos G, et al. LAMP kit for diagnosis of non-falciparum malaria in Plasmodium ovale infected patients. Malar J 2017, 16:20.
97. Piera KA, Aziz A, William T, Bell D, González IJ, Barber BE, et al. Detection of Plasmodium knowlesi, Plasmodium falciparum and Plasmodium vivax using loop-mediated isothermal amplification (LAMP) in a co-endemic area in Malaysia. Malar J 2017, 16:29.
98. Coutrier FN, Tirta YK, Cotter C, Zarlinda I, González IJ, Schwartz A, et al. Laboratory challenges of Plasmodium species identification in Aceh Province, Indonesia, a malaria elimination setting with newly discovered P. knowlesi. PLoS Negl Trop Dis 2018, 12:e0006924.
99. Nolasco O, Infante B, Contreras-Mancilla J, Incardona S, Ding XC, Gamboa D, et al. Diagnosis of Plasmodium vivax by Loop-Mediated Isothermal Amplification in Febrile Patient Samples from Loreto, Perú. Am J Trop Med Hyg 2020.

Evaluation of cost effectiveness [1]

100. Zelman BW, Baral R, Zarlinda I, Coutrier FN, Sanders KC, Cotter C, et al. Costs and cost-effectiveness of malaria reactive case detection using loop-mediated isothermal amplification compared to microscopy in the low transmission setting of Aceh Province, Indonesia. Malar J 2018, 17:220.

LAMP application

Prevalence surveys [9]

101. Katrak S, Day N, Ssemmondo E, Kwarisiima D, Midekisa A, Greenhouse B, et al. Community-wide Prevalence of Malaria Parasitemia in HIV-Infected and Uninfected Populations in a High-Transmission Setting in Uganda. J Infect Dis 2016, 213:1971-8. (Loopamp™)
102. Rek J, Katrak S, Obasi H, Nayebare P, Katureebe A, Kakande E, et al. Characterizing microscopic and submicroscopic malaria parasitaemia at three sites with varied transmission intensity in Uganda. Malar J 2016, 15:470. (Loopamp™)
103. Dlamini N, Hsiang MS, Ntshalintshali N, Pindolia D, Allen R, Nhlabathi N, et al. Low-Quality Housing Is Associated With Increased Risk of Malaria Infection: A National Population-Based Study From the Low Transmission Setting of Swaziland. Open Forum Infect Dis 2017, 4:ofx071. (Loopamp™)
104. Smith JL, Auala J, Tambo M, Haindongo E, Katokele S, Uusiku P, et al. Spatial clustering of patent and sub-patent malaria infections in northern Namibia: Implications for surveillance and response strategies for elimination. PLoS One 2017, 12:e0180845. (Loopamp™)
105. Katrak S, Nayebare P, Rek J, Arinaitwe E, Nankabirwa JI, Kamya M, et al. Clinical consequences of submicroscopic malaria parasitaemia in Uganda. Malar J 2018, 17:67. (Loopamp™)
106. McCreesh P, Mumbengegwi D, Roberts K, Tambo M, Smith J, Whittemore B, et al. Subpatent malaria in a low transmission African setting: a cross-sectional study using rapid diagnostic testing (RDT) and loop-mediated isothermal amplification (LAMP) from Zambezi region, Namibia. Malar J 2018, 17:480. (Loopamp™)
107. Sattabongkot J, Suansomjit C, Nguitragool W, Sirichaisinthop J, Warit S, Tiensuwan M, et al. Prevalence of asymptomatic Plasmodium infections with sub-microscopic parasite densities in the northwestern border of Thailand: a potential threat to malaria elimination. Malar J 2018, 17:329. (other)
108. Nankabirwa JI, Briggs J, Rek J, Arinaitwe E, Nayebare P, Katrak S, et al. Persistent Parasitemia Despite Dramatic Reduction in Malaria Incidence After 3 Rounds of Indoor Residual Spraying in Tororo, Uganda. J Infect Dis 2019, 219:1104-11. (Loopamp™)
109. Lufungulo Bahati Y, Delanghe J, Bisimwa Balaluka G, Sadiki Kishabongo A, Philippé J. Asymptomatic Submicroscopic Plasmodium Infection Is Highly Prevalent and Is Associated with Anemia in Children Younger than 5 Years in South Kivu/Democratic Republic of Congo. Am J Trop Med Hyg 2020, 102:1048-55. (Illumigene)

LAMP in pregnancy [12]

110. Prahl M, Jagannathan P, McIntyre TI, Auma A, Farrington L, Wamala S, et al. Timing of in utero malaria exposure influences fetal CD4 T cell regulatory versus effector differentiation. Malar J 2016, 15:497. (Loopamp™)
111. Kapisi J, Kakuru A, Jagannathan P, Muhindo MK, Natureeba P, Awori P, et al. Relationships between infection with Plasmodium falciparum during pregnancy, measures of placental malaria, and adverse birth outcomes. Malar J 2017, 16:400. (Loopamp™)
112. Tegegne B, Getie S, Lemma W, Mohon AN, Pillai DR. Performance of loop-mediated isothermal amplification (LAMP) for the diagnosis of malaria among malaria suspected pregnant women in Northwest Ethiopia. Malar J 2017, 16:34. (Loopamp™)
113. Vásquez AM, Zuluaga L, Tobón A, Posada M, Vélez G, González IJ, et al. Diagnostic accuracy of loop-mediated isothermal amplification (LAMP) for screening malaria in peripheral and placental blood samples from pregnant women in Colombia. Malar J 2018, 17:262. (Loopamp™)

114. Ategeka J, Kakuru A, Kajubi R, Wasswa R, Ochokoru H, Arinaitwe E, et al. Relationships Between Measures of Malaria at Delivery and Adverse Birth Outcomes in a High-Transmission Area of Uganda. J Infect Dis 2020, 222:863-70. (Loopamp™)
115. Tadesse G, Kamaliddin C, Doolan C, Amarasekara R, Legese R, Mohon AN, et al. Active case detection of malaria in pregnancy using loop-mediated amplification (LAMP): a pilot outcomes study in South West Ethiopia. Malar J 2020, 19:305. (Illumigene)
116. Tran EE, Cheeks ML, Kakuru A, Muhindo MK, Natureeba P, Nakalembe M, et al. The impact of gravidity, symptomatology and timing of infection on placental malaria. Malar J 2020, 19:227. (Loopamp™)
117. Unwin VT, Ahmed R, Noviyanti R, Puspitasari AM, Utami RAS, Trianty L, et al. Use of a highly-sensitive rapid diagnostic test to screen for malaria in pregnancy in Indonesia. Malar J 2020, 19:28. (Loopamp™)
118. Vásquez AM, Vélez G, Medina A, Serra-Casas E, Campillo A, Gonzalez IJ, et al. Evaluation of highly sensitive diagnostic tools for the detection of P. falciparum in pregnant women attending antenatal care visits in Colombia. BMC Pregnancy Childbirth 2020, 20:440. (Loopamp™)
119. Kakuru A, Jagannathan P, Muhindo MK, Natureeba P, Awori P, Nakalembe M, Opira B, Olwoch P, Ategeka J, Nayebare P, et al: Dihydroartemisinin-Piperaquine for the Prevention of Malaria in Pregnancy. *N Engl J Med* 2016, 374:928-939. (Loopamp™)
120. Ahmed R, Poespoprodjo JR, Syafruddin D, Khairallah C, Pace C, Lukito T, Maratina SS, Asih PBS, Santana-Morales MA, Adams ER, et al: Efficacy and safety of intermittent preventive treatment and intermittent screening and treatment versus single screening and treatment with dihydroartemisinin-piperaquine for the control of malaria in pregnancy in Indonesia: a cluster-randomised, open-label, superiority trial. *Lancet Infectious Diseases* 2019, 19:973-987. (Loopamp™)
121. Kajubi R, Ochieng T, Kakuru A, Jagannathan P, Nakalembe M, Ruel T, Opira B, Ochokoru H, Ategeka J, Nayebare P, et al: Monthly sulfadoxine-pyrimethamine versus dihydroartemisinin-piperaquine for intermittent preventive treatment of malaria in pregnancy: a double-blind, randomised, controlled, superiority trial. *Lancet* 2019, 393:1428-1439. (Loopamp™)

LAMP in Travelers [11]

122. Polley SD, González IJ, Mohamed D, Daly R, Bowers K, Watson J, et al. Clinical evaluation of a loop-mediated amplification kit for diagnosis of imported malaria. J Infect Dis 2013, 208:637-44. (Loopamp™)
123. Marti H, Stalder C, González IJ. Diagnostic accuracy of a LAMP kit for diagnosis of imported malaria in Switzerland. Travel Med Infect Dis 2015, 13:167-71. (Loopamp™)
124. Mohon AN, Lee LD, Bayih AG, Folefoc A, Guelig D, Burton RA, et al. NINA-LAMP compared to microscopy, RDT, and nested PCR for the detection of imported malaria. Diagn Microbiol Infect Dis 2016, 85:149-53. (Loopamp™)
125. De Koninck AS, Cnops L, Hofmans M, Jacobs J, Van den Bossche D, Philippé J. Diagnostic performance of the loop-mediated isothermal amplification (LAMP) based illumigene(®) malaria assay in a non-endemic region. Malar J 2017, 16:418. (Illumigene)
126. Rypien C, Chow B, Chan WW, Church DL, Pillai DR. Detection of Plasmodium Infection by the illumigene Malaria Assay Compared to Reference Microscopy and Real-Time PCR. J Clin Microbiol 2017, 55:3037-45. (Illumigene)
127. Ponce C, Kaczorowski F, Perpoint T, Miailhes P, Sigal A, Javouhey E, et al. Diagnostic accuracy of loop-mediated isothermal amplification (LAMP) for screening patients with imported malaria in a non-endemic setting. Parasite 2017, 24:53. (Illumigene)

128. Cheaveau J, Nguyen H, Chow B, Marasinghe D, Mohon AN, Yuan H, et al. Clinical Validation of a Commercial LAMP Test for Ruling out Malaria in Returning Travelers: A Prospective Diagnostic Trial. Open Forum Infect Dis 2018, 5:ofy260. (Illumigene)
129. Frickmann H, Hinz R, Rojak S, Bonow I, Ruben S, Wegner C, et al. Evaluation of automated loop-mediated amplification (LAMP) for routine malaria detection in blood samples of German travelers - A cross-sectional study. Travel Med Infect Dis 2018, 24:25-30. (Illumigene)
130. Burdino E, Calleri G, Ghisetti V. Added value of loop-mediated isothermal amplification technology (LAMP) in real life for the diagnosis of malaria in travellers. J Travel Med 2019, 26. (Illumigene)
131. Hartmeyer GN, Hoegh SV, Skov MN, Kemp M. Use of Loop-Mediated Isothermal Amplification in a Resource-Saving Strategy for Primary Malaria Screening in a Non-Endemic Setting. Am J Trop Med Hyg 2019, 100:566-71. (Illumigene)
132. Charpentier E, Benichou E, Pagès A, Chauvin P, Fillaux J, Valentin A, et al. Performance evaluation of different strategies based on microscopy techniques, rapid diagnostic test and molecular loop-mediated isothermal amplification assay for the diagnosis of imported malaria. Clin Microbiol Infect 2020, 26:115-21. (Illumigene)

Other areas of LAMP application

LAMP for molecular markers of drug resistance [9]

133. Abdul-Ghani R. Towards rapid genotyping of resistant malaria parasites: could loop-mediated isothermal amplification be the solution? Malar J 2014, 13:237.
134. Chahar M, Mishra N, Anvikar A, Dixit R, Valecha N. Establishment and application of a novel isothermal amplification assay for rapid detection of chloroquine resistance (K76T) in Plasmodium falciparum. Sci Rep 2017, 7:41119.
135. Yongkiettrakul S, Kampeera J, Chareanchim W, Rattanajak R, Pornthanakasem W, Kiatpathomchai W, et al. Simple detection of single nucleotide polymorphism in Plasmodium falciparum by SNP-LAMP assay combined with lateral flow dipstick. Parasitol Int 2017, 66:964-71.
136. Chahar M, Anvikar A, Dixit R, Valecha N. Evaluation of four novel isothermal amplification assays towards simple and rapid genotyping of chloroquine resistant Plasmodium falciparum. Exp Parasitol 2018, 190:1-9.
137. Imai K, Tarumoto N, Runtuwene LR, Sakai J, Hayashida K, Eshita Y, et al. An innovative diagnostic technology for the codon mutation C580Y in kelch13 of Plasmodium falciparum with MinION nanopore sequencer. Malar J 2018, 17:217.
138. Malpartida-Cardenas K, Rodriguez-Manzano J, Yu LS, Delves MJ, Nguon C, Chotivanich K, et al. Allele-Specific Isothermal Amplification Method Using Unmodified Self-Stabilizing Competitive Primers. Anal Chem 2018, 90:11972-80.
139. Mohon AN, Menard D, Alam MS, Perera K, Pillai DR. A Novel Single-Nucleotide Polymorphism Loop Mediated Isothermal Amplification Assay for Detection of Artemisinin-Resistant Plasmodium falciparum Malaria. Open Forum Infect Dis 2018, 5:ofy011.
140. Chahar M, Anvikar A, Valecha N. Development and Evaluation of a Novel HNB Based Isothermal Amplification Assay for Fast Detection of Pyrimethamine Resistance (S108N) in Plasmodium falciparum. Int J Environ Res Public Health 2019, 16.
141. Malpartida-Cardenas K, Miscourides N, Rodriguez-Manzano J, Yu LS, Moser N, Baum J, et al. Quantitative and rapid Plasmodium falciparum malaria diagnosis and artemisinin-resistance detection using a CMOS Lab-on-Chip platform. Biosens Bioelectron 2019, 145:111678.

LAMP for Pf gametocyte detection [2]

142. Buates S, Bantuchai S, Sattabongkot J, Han ET, Tsuboi T, Udomsangpetch R, et al. Development of a reverse transcription-loop-mediated isothermal amplification (RT-LAMP) for clinical detection of Plasmodium falciparum gametocytes. Parasitol Int 2010, 59:414-20.
143. Essangui E, Eboumbou Moukoko CE, Nguedia N, Tchokwansi M, Banlanjo U, Maloba F, et al. Demographical, hematological and serological risk factors for Plasmodium falciparum gametocyte carriage in a high stable transmission zone in Cameroon. PLoS One 2019, 14:e0216133.

LAMP in the evaluation of new diagnostic tools [2]

144. Tambo M, Mwinga M, Mumbengegwi DR. Loop-mediated isothermal amplification (LAMP) and Polymerase Chain Reaction (PCR) as quality assurance tools for Rapid Diagnostic Test (RDT) malaria diagnosis in Northern Namibia. PLoS One 2018, 13:e0206848. (Loopamp™?)
145. Lalremruata A, Nguyen TT, McCall MBB, Mombo-Ngoma G, Agnandji ST, Adegnika AA, et al. Recombinase Polymerase Amplification and Lateral Flow Assay for Ultrasensitive Detection of Low-Density Plasmodium falciparum Infection from Controlled Human Malaria Infection Studies and Naturally Acquired Infections. J Clin Microbiol 2020, 58. (Loopamp™)
146. Pomari E, Silva R, Moro L, La Marca G, Perandin F, Verra F, et al. Droplet Digital PCR for the Detection of Plasmodium falciparum DNA in Whole Blood and Serum: A Comparative Analysis with Other Molecular Methods. Pathogens 2020, 9. (Illumigene)

LAMP in hrp2/3 deletion assessments [2]

147. Thomson R, Beshir KB, Cunningham J, Baiden F, Bharmal J, Bruxvoort KJ, et al. pfhrp2 and pfhrp3 Gene Deletions That Affect Malaria Rapid Diagnostic Tests for Plasmodium falciparum: Analysis of Archived Blood Samples From 3 African Countries. J Infect Dis 2019, 220:1444-52. (Not specified)
148. Pasquier G, Azoury V, Sasso M, Laroche L, Varlet-Marie E, Houzé S, et al. Rapid diagnostic tests failing to detect infections by Plasmodium falciparum encoding pfhrp2 and pfhrp3 genes in a non-endemic setting. Malar J 2020, 19:179. (Illumigene)
